# Triglyceride Can Predict the Discordance between QCT and DXA Screening for BMD in Old Female Patients

**DOI:** 10.1155/2020/8898888

**Published:** 2020-12-27

**Authors:** Dongjiang Xu, Ke di Wang, Jianhong Yang

**Affiliations:** ^1^Savaid Medical School, University of Chinese Academy of Sciences, Beijing 100049, China; ^2^Department of Clinical Laboratory Medicine, Beijing Jishuitan Hospital, Beijing 100035, China; ^3^Department of Clinical Laboratory Medicine, Beijing Friendship Hospital, Capital Medical University, Beijing, China

## Abstract

**Purpose:**

This study is aimed at exploring which indicator can predict the discordance between DXA and QCT.

**Methods:**

192 female patients who took BMD screening tests by QCT and DXA were recruited, and the biomarkers were analyzed to study the relationship between the biomarkers and the discordance of two BMD screening methods.

**Results:**

There are 42, 78, and 72 female patients in the normal, osteopenia, and osteoporosis groups defined by DXA and 6, 54, and 132 female patients in the corresponding group defined by QCT. DXA was less sensitive than QCT. Cholesterol (CHO) and triglyceride (TG) were all negatively correlated with the discordance between these two methods. When TG > 0.89 mmol/L, the QCT result would be the same as the DXA's; otherwise, there should be discordance between QCT and DXA.

**Conclusions:**

Triglyceride can be used to predict the discordance between QCT and DXA, and clinicians can evaluate patients' DXA results based on patient triglyceride or cholesterol results as a supplement to QCT results.

## 1. Introduction

Osteoporosis is a common metabolic bone disorder characterized by low bone mineral mass and microarchitectural deterioration of bone tissue resulting in enhanced bone fragility and an increased risk of fragility fracture [1]. Fractures caused by osteoporosis are often associated with significant morbidity and mortality. Osteoporotic hip fractures and vertebral fractures have become a major public problem following the increasing elderly population. It is very important to pay much attention to the screening for osteoporosis. Now, the clinical gold standard for diagnosing osteoporosis is areal bone mineral density (aBMD), measured by dual-energy X-ray absorptiometry (DXA, previously DEXA) [2]. Two X-ray beams, with different energy levels, are pointed at the patient's bones. When soft tissue absorption is subtracted out, the BMD can be determined from the absorption of each beam by bone. DXA is the most widely available and commonly used due to its reproducibility, negligible radiation dose, and reliable pediatric reference data. Some variables are suggested to confound the interpretation of BMD as measured by DXA, such as bone size, body lipids, sex, and age [3, 4]. Another technology for BMD is quantitative computed tomography (QCT) [5]. QCT measures BMD using a standard X-ray computed tomography (CT) scanner with a calibration standard to convert Hounsfield Units (HU) of the CT image to bone mineral density values at the lumbar spine and hip.

CT is the first choice for patients with traumatic fracture and spinal degenerative disease to detect bone status. The patients were scanned with the calibration phantom beneath the back while performing a spine CT scan. Doctors can evaluate QCT bone mineral density data of patients without DXA detection. However, each of the screening technology has its diagnosis category [6, 7]. There are many studies on the discordance between the DXA and QCT screening results [8, 9]. Woodson put the discordance classified by degrees into either minor or major categories [10]. Until now, scientists have focused on the different principles between the two technologies to interpret the discordance. No article is designed to study the relationship between the biomarkers and the discordance of BMD screening tests by DXA and QCT. Our article is aimed at exploring whether the biomarkers play a great role in the discordance between the two BMD screening methods to find the serological indicators which can make up for the difference between DXA and QCT results.

## 2. Material and Methods

### 2.1. Source Population

All participants were selected consecutively from the Cadre Health Care Department of Beijing Jishuitan Hospital between October 2018 and January 2019. The exclusion criteria included (a) diagnosis of malignant tumor and severe heart, liver, or kidney diseases such as cirrhosis and dialysis; (b) compression fracture of lumbar vertebrae which should affect measurement results in two years; (c) diagnosis of pituitary, thyroid, parathyroid, adrenal, and gonadal diseases; (d) obese patients; and (e) history of drug therapy (especially antiosteoporosis drugs, estrogen replacement therapy, and glucocorticoids). This study is a collection or study of previously archived data, documents, records, pathological specimens, or diagnostic specimens, and these are public resources, so the ethics committee of our institution approved the study, and written consent is not required. Anthropometric characters such as height (cm) and weight (kg) were measured by the nurses, and body mass index (BMI) was calculated as weight divided by height squared.

### 2.2. Serum Collection and Biomarker Analyzation

Serum samples were collected at 6 a.m. after overnight fasting (8 h at least) in the ward nurse station and transported to the laboratory department. In the laboratory department, all the samples were allowed to clot for 30 min at room temperature then centrifuged at 2500g relative centrifugal force (RCF) for 15 minutes and analyzed on the same day.

The serum samples were analyzed following the standard operation procedure of the laboratory department; serum lipid metabolism indexes (Merit Choice Bioengineering Co., Ltd., Beijing) were chosen to be tested including cholesterol (CHO), triglyceride (TG), high-density lipoprotein cholesterol (HDL-C), and low-density lipoprotein cholesterol (LDL-C) levels.

### 2.3. DXA Performance

Bone mass was measured densitometrically at the anterior-posterior lumbar spine (L2–L4) and left hip (femoral neck, trochanter, and total hip) using a GE Lunar iDXA scanner (GE Healthcare, Madison, WI); then, the lowest *T*-score value of the lumbar vertebrae, femoral neck, and total hip was used to evaluate the bone condition. The patients were classified into three groups based on the World Health Organization (WHO) classification of BMD for the diagnosis of osteoporosis: normal (*T*‐score ≥ −1.0), osteopenia (*T*-score between −1.1 and −2.5), and osteoporosis (*T*‐score ≤ −2.5).

### 2.4. QCT Examination

QCT (Toshiba (Tokyo, Europe) Aquilion 16-slice computed tomography unit) was used for bone density measurement of L1-L4 in the lumbar spine. All the patients were scanned in the supine position, with a solid QCT phantom (Mindways Software Inc., Austin, TX, USA) placed below them, on the midline in the thoracolumbar region. All the lumbar spine images were independently reviewed by two experienced radiologists on a three-dimensional workstation to assess the presence of vertebral fractures. Whenever they disagreed on the results, they would reach an agreement after discussion. The patients were classified into three groups based on the diagnostic criteria of the American College of Radiology: osteoporosis, BMD < 80 mg/cm^3^; osteopenia, 80 mg/cm^3^ ≤ BMD ≤ 120 mg/cm^3^; and normal, BMD > 120 mg/cm^3^.

### 2.5. Differences between the Results of the Two Technologies

Concordance was defined as present when the screening results of two methods placed the patient in the same diagnostic class. Minor discordance was defined as present when the difference between the two methods is no more than one class. Major discordance was defined when one method screening result is normal and the other is osteoporotic.

### 2.6. Statistical Analysis

Means ± standard deviation (SD) was used for continuous variables. The differences in the clinical characteristics of patients were determined with the *χ*^2^ test for categorical variables. One-way ANOVA was performed to determine the significances between different groups. Partial correlations analysis was performed to evaluate the correlations between biomarkers and the categorical variables when they were adjusted by BMI and age. A binary logistic regression model was performed to assess the importance of included variables by calculating the partial correlation coefficient. A *p* value < 0.05 was considered statistically significant. All analyses were performed using SPSS version 24 (IBM SPSS, IBM Corp., NY, USA).

## 3. Results

### 3.1. Patient Clinical Characteristics

The patient characteristics are presented in Table 1. This retrospective study included 192 female patients with a mean age of 76.81 ± 8.89 years and a BMI of 22.64 ± 3.45 kg/m^2^.

### 3.2. Diagnostic Results of DXA versus QCT for 192 Participants

As shown in Table 2, 42, 78, and 72 female patients were included in the normal, osteopenia, and osteoporosis groups defined by DXA and 6, 54, and 132 female patients were enrolled in the normal, osteopenia, and osteoporosis groups defined by QCT. The intragroup detection rates for QCT versus DXA were significantly different (*p* < 0.01), with QCT detecting osteoporosis more frequently than DXA did.

### 3.3. Comparison of Biomarkers among the Concordance, Minor Discordance, and Major Discordance Groups

The associations and comparison of the biomarkers among the three groups are shown in Table 3. There are 114, 60, and 18 female patients in the concordance, minor discordance, and major discordance groups. CHO, TG, HDL, and LDL showed significant differences between the three discordance groups.

### 3.4. Bivariate Correlation and Binary Logistic Regression Analyses

We divided the classifications of the two methods into the concordance group (group 1) and the discordance group (group 2); CHO (*r* = −0.267, *p* < 0.01), LDL (*r* = −0.189, *p* < 0.01), and TG (*r* = −0.376, *p* < 0.01) showed a significantly negative relationship with the two groups (Table 4). There was a correlation between these three items, but TG got the largest correlation coefficient, so we took a binary logistic regression analysis on TG with the two groups. The regression model was shown as follows: log(group) = 1.346 − 1.505∗TG.

When TG > 0.89 mmol/L, we can judge that the QCT result is the same as the DXA's; otherwise, there should be discordance between QCT and DXA.

## 4. Discussion

Many studies have found convincing evidence that bone measurement tests are accurate for predicting osteoporotic fractures in old people. The most commonly used test is DXA of the hip and lumbar spine and QCT scanned in the supine position, with a solid QCT phantom.

However, we found significant differences in the classification using QCT and DXA, with the QCT measurements indicating higher percentages of both osteopenia and osteoporosis. We tried to analyze which serological indicators can help to judge the discordance between the two methods.

Blood lipid markers were more prominent in the elderly female population when we tried to find out the reasons for the differences between DXA and QCT screening for osteoporosis. The grading of osteoporosis determined by the two methods, including the discordance degree between the two methods, was significantly correlated with blood lipids. The relationship between blood lipid metabolism and bone mineral density has been widely studied in the general population; there are some potential mechanisms of serum lipid on bone metabolism. Oxysterols should be removed by HDL-C from peripheral tissues, and osteoblasts' differentiation is inhibited by LDL oxidation products, so cholesterol, HDL-C, and LDL-C can direct progenitor MSCs (mesenchymal stem cells) to undergo lipogenic instead of osteogenic differentiation and induce RANKL- (receptor activator of nuclear factor-kB ligand-) dependent osteoclastic differentiation, and our results also showed their negative effects on osteogenic differentiation [11, 12]. Trimpou et al. [13] observed necrosis of the femoral head under an electron microscope and found that the number and size of fat cells increased significantly, indicating that lipid metabolism may play an important role in the formation of bone geometry. In the meantime, it is reported that [14] fatty acids, phospholipids, and several endogenous metabolites play a key role in the homeostasis level of bones. For example, by acting on the survival and function of bone cells, participating in the process of bone mineralization, and even regulating various key signaling pathways. This may explain that lipid metabolism indicators help predict the inconsistency between QCT and DXA results.

Overall, from this study, we conclude that TG was an important factor for the prediction of the discordance between DXA and QCT, so we suggest that clinicians should review actual scan images and biomarker data, instead of relying solely on the impression of the report, to pinpoint errors and accurately interpret DXA and QCT screening results. They should keep the idea that biomarkers may play a great role in the difference between DXA and QCT screening for osteoporosis in old female patients. It is an opportunity to promote awareness and education about the critical need to better understand this technology and assure high-quality DXA acquisition, analysis, and interpretation to optimize patient care.

## 5. Conclusions

According to the actual situation of patients, doctors should comprehensively analyze the bone mineral density of patients; especially for patients with traumatic fracture and spinal degenerative disease, the QCT data of patients should be combined with TG to judge whether there is a difference between the results of DXA, and the operation mode and prognosis plan should be adjusted according to the actual situation.

## Figures and Tables

**Table 1:** Demographics of patients.

Variables	Female patients (*n* = 192)	
	Mean	SD
*Character*		
Age (year)	76.81	8.89
Height (cm)	157.6	6.22
Weight (kg)	57.28	10.82
Body mass index (kg/m^2^)	22.64	3.45
*Lipid metabolism index*		
Cholesterol (mmol/L)	4.62	0.55
Triglyceride (mmol/L)	1.77	[1.59, 1.97]
High-density lipoprotein cholesterol (mmol/L)	1.47	0.18
Low-density lipoprotein cholesterol (mmol/L)	2.32	0.46
*Bone mineral density*		
Dual-energy X-ray absorptiometry (DXA)	-1.78	1.34
Quantitative computed tomography (QCT)	59.79	30.57

**Table 2:** Diagnostic results of DXA versus QCT for 192 participants.

Methods	Osteoporosis (%)	Osteopenia (%)	Normal (%)
DXA	72 (37.5)	78 (40.6)	42 (21.9)
QCT	132 (68.8)	54 (28.1)	6 (3.1)
Pearson chi square	49.01		
*p*	<0.001		

DXA: dual-energy X-ray absorptiometry; QCT: quantitative computed tomography.

**Table 3:** Comparison of biomarkers among the concordance, minor discordance, and major discordance groups.

D-group	Concordance (*n* = 114)		Minor discordance (*n* = 60)		Major discordance (*n* = 18)		*F*	*p*
	Mean	SD	Mean	SD	Mean	SD		
Age (year)	77.5	10.08	76.1	7.46	75.38	2.23	0.674	0.511
BMI (kg/m^2^)	22.81	4.11	24.83	4.61	18.09	0.71	14.21	0.001^∗∗^
CHO (mmol/L)	4.72	1.29	3.98	1.13	4.19	1.07	7.406	0.001^∗∗^
TG (mmol/L)	1.67	1.0	1.00	0.34	0.89	0.49	15.615	0.001^∗∗^
HDL-C (mmol/L)	1.36	0.36	1.39	0.37	1.70	0.18	5.084	0.007^∗∗^
LDL-C (mmol/L)	2.42	0.86	2.08	0.70	2.23	0.68	3.712	0.026^∗^

BMI: body mass index; CHO: cholesterol; TG: triglyceride; HDL-C: high-density lipoprotein cholesterol; LDL-C: low-density lipoprotein cholesterol; ^∗^*p* < 0.05; ^∗∗^*p* < 0.01.

**Table 4:** Bivariate correlation analyses.

		Group	CHO	TG	HDL	LDL
Group	Pearson correlation	1	-0.267	-0.376	0.111	-0.189
	*p*		0.001^∗∗^	0.001^∗∗^	0.127	0.009^∗∗^
CHO	Pearson correlation	-0.267	1	0.460	0.420	0.963
	*p*	0.001^∗∗^		0.001^∗∗^	0.001^∗∗^	0.001^∗∗^
TG	Pearson correlation	-0.376	0.460	1	-0.127	0.360
	*p*	0.001^∗∗^	0.001^∗∗^		0.08	0.001^∗∗^
HDL	Pearson correlation	0.111	0.420	-0.127	1	0.464
	*p*	0.127	0.001^∗∗^	0.08		0.001^∗∗^
LDL	Pearson correlation	-0.189	0.963	0.360	0.464	1
	*p*	0.009^∗∗^	0.001^∗∗^	0.001^∗∗^	0.001^∗∗^	

CHO: cholesterol; TG: triglyceride; HDL-C: high-density lipoprotein cholesterol; LDL-C: low-density lipoprotein cholesterol; ^∗^*p* < 0.05; ^∗∗^*p* < 0.01.

## Data Availability

The data are available as requested.
